# Fostering incidental experiences of nature through green infrastructure planning

**DOI:** 10.1007/s13280-017-0920-z

**Published:** 2017-04-25

**Authors:** Thomas H. Beery, Christopher M. Raymond, Marketta Kyttä, Anton Stahl Olafsson, Tobias Plieninger, Mattias Sandberg, Marie Stenseke, Maria Tengö, K. Ingemar Jönsson

**Affiliations:** 1 School of Education and Environment, Kristianstad University, 291 88 Kristianstad, Sweden; 2 Department of Landscape Architecture, Planning and Management, Swedish University of Agricultural Sciences, P.O. Box 58, Alnarp, Sweden; 3 Aalto University, Department of Built Environment, P.O. Box 14100, 00076 Aalto, Finland; 4 Department of Geosciences and Natural Resource Management, University of Copenhagen, Rolighedsvej 23, 1958 Frederiksberg C, Denmark; 5 Unit for Human Geography, Department of Economy and Society, University of Gothenburg, P.O. Box 625, 405 30 Göteborg, Sweden; 6 Stockholm Resilience Center, Stockholm, Sweden; 7 Enviroconnect, PO Box 190, Stirling, SA 5152 Australia

**Keywords:** Extinction of experience, Human well-being, Incidental nature experience, Intentional nature experience, Nudging, Redirection of attention

## Abstract

Concern for a diminished human experience of nature and subsequent decreased human well-being is addressed via a consideration of green infrastructure’s potential to facilitate unplanned or incidental nature experience. Incidental nature experience is conceptualized and illustrated in order to consider this seldom addressed aspect of human interaction with nature in green infrastructure planning. Special attention has been paid to the ability of incidental nature experience to redirect attention from a primary activity toward an unplanned focus (in this case, nature phenomena). The value of such experience for human well-being is considered. The role of green infrastructure to provide the opportunity for incidental nature experience may serve as a nudge or guide toward meaningful interaction. These ideas are explored using examples of green infrastructure design in two Nordic municipalities: Kristianstad, Sweden, and Copenhagen, Denmark. The outcome of the case study analysis coupled with the review of literature is a set of sample recommendations for how green infrastructure can be designed to support a range of incidental nature experiences with the potential to support human well-being.

## Introduction

Over half of the global human population now lives in urban areas and by 2050 this proportion is expected to exceed 90% for developed countries (United Nations Department of Economic and Social Affairs 2014). This growth and shift from rural to urban living is associated with a decrease in human population living with direct and accessible exposure to green and blue environments (Skår and Krogh 2009; Elmqvist et al. 2013). One outcome from this trend is a concern that many people today do not have adequate opportunity to interact with nature^1^ in outdoor settings at levels available to previous generations. This phenomenon has been referred to as an *extinction of experience* (Nabhan and St. Antoine 1993; Pyle 1993; Thomashow 2002; Miller 2005; Krasny 2015; Soga and Gaston 2016) and has been described as resulting in a decline in ways of learning and thinking about the natural world (Thomashow 2002). The phrase was used by Pyle (1993) to contrast his own rich childhood nature experience which he described as coming not from pristine wilderness, but rather from a proximate and untamed suburban nature. In Pyle’s case, it was a ditch in his neighborhood, a part of the High Line Canal built outside Denver for irrigation purposes, where he found access to freely explore nature (Pyle 1993). Krasny (2015) reminds us that these opportunities to counter the extinction of experience and interact with nature may happen in a range of important places, from far-flung wilderness to places proximate and urban, from city parks to national parks.

In support of increased connection to nature, over 40 years of research has provided compelling arguments showing that experiences of nature in green areas are linked to a breadth of positive human well-being outcomes. These include improved physical health, improved mental well-being, greater social well-being, and the promotion of positive health behaviors such as physical activity (Maller et al. 2008; Keniger et al. 2013; Sandifer et al. 2015; Shanahan et al. 2016). These links between nature experience and well-being are now recognized in frameworks for the assessment of impacts of nature-based solutions in urban areas (Raymond et al. 2017) and in a roadmap for health–social–nature synergies (ten Brink et al. 2016). They are also recognized globally in international science–policy platforms including the Intergovernmental Panel on Biodiversity and Ecosystem Services or IPBES (Díaz et al. 2015; Pascual et al. 2017). In response, and in conjunction with acknowledgement of ecosystem values and functions, cities across the world are making investments into green infrastructure to support a wide variety of outcomes including human well-being (Hammer et al. 2011). There is current interest in the potential links between nature, values, and health/well-being with connection to nature or experience with nature (Capaldi et al. 2015; Shanahan et al. 2016). Many of these links are focused on the benefits from *intentional* experience, defined as experiencing or being in nature through direct intention (Keniger et al. 2013). In this perspective article, however, we make the case for considering unintentional, or *incidental*, nature experience, and show how it can be done in the context of green infrastructure planning. We use specific examples from two Nordic urban areas, Copenhagen, Denmark, and Kristianstad, Sweden, to illustrate the potential of green infrastructure planning to facilitate incidental nature experience. Specifically, we

1. compare different forms of intentional and incidental nature experience, and the potential for transitions among them;
2. showcase how green infrastructure design can accommodate a range of intentional and incidental nature experiences using cases from Kristianstad, Sweden, and Copenhagen, Denmark; and
3. make recommendations about how green infrastructure could be designed to support a range of intentional and incidental direct nature experiences.

## Background

### Green infrastructure

Green infrastructure is defined as “…an interconnected network of green space that conserves natural ecosystem values and functions and provides associated benefits to human populations” (Benedict and McMahon 2002, p. 12). This network of urban nature including forests, wetlands, parks, grasslands, trees, flower beds, green court yards, and green roofs is the biophysical green of a green–gray continuum (Mell 2013). Such a network corresponds to the conceptualization of urban nature in the recent strategy of Copenhagen and reflects decades of green space and green infrastructure planning in Nordic cities (Copenhagen 2015a). In the EU Green Infrastructure Strategy 2013–2020, there is recognition that green infrastructure can provide a range of biodiversity as well as social and cultural outcomes in terms of human well-being and life quality (European Commission 2013). Addressing multiple values is frequently discussed in the context of multi-functional green infrastructure, described by Sandifer et al. (2015) as putting “…human health and well-being at the center…” thus facilitating human interaction with nature and ensuring that “…people are surrounded by and have access to biologically diverse natural habitats” (p. 12). This approach to green infrastructure is consistent with the idea of biophilic cities, where frequent and qualitative contact with nature as a daily experience is supported (Beatley 2011). We argue that daily living activity (for example, mobility for work, school, and basic needs) within a network of green infrastructure provides important intentional as well as incidental nature interaction opportunity.

### Intentional and incidental nature experiences

Interactions between people and nature have been classified into three broad categories that are useful for a deeper consideration of nature experience (Keniger et al. 2013):

- Indirect: experiencing nature while not being present in it.
- (Direct) Intentional: experiencing or being in nature through direct intention.
- (Direct) Incidental: experiencing nature as a by-product of another activity.

This paper will not address indirect nature experience, but will instead focus on direct experiences (see Table 1). Specifically, the unique quality of incidental experience and consideration of the interaction between incidental and intentional nature experience will be explored.

**Table 1:** Examples of direct daily nature experience: Intentional, incidental, and the interaction between the intentional and incidental

	Intentional nature experience Planned encounters	Interaction between intentional and incidental nature experience	Incidental nature experience Unplanned encounters
Action, behavior, or situation^a^	Wildlife observation in a park Gardening in one’s yard Stargazing on a dark night Collecting shells and rocks on a beach Walking outdoors during a snowstorm Climbing a rock cliff	Picking berries in a forest and discovering tracks from a wild animal Eating lunch outdoors to enjoy the weather and noting early autumn color change Mushroom foraging along a wooded path and being surprised by the unexpected movement of a snake	Noticing a colorful sunset while walking to the grocery store Getting wet during a sudden downpour while biking to work Appreciating fragrance from blooming trees while attending to outdoor household chores Hearing an interesting bird song while waiting for the bus

^a^These experiences are not exclusively positive, and some nature experiences may be perceived as positive by some and negative by others. For example, a dark night providing stargazing opportunity could be perceived as a negative if fears about personal safety are associated with dark night nature experiences. The intent in this perspective, however, is to focus on positive opportunity

Incidental nature experience can be described as sudden awareness of previously unnoticed, yet regular natural features that come to one’s attention in unplanned or unexpected ways, such as the surprise discovery of both the sharpness of blackberry thorns and the ripeness of the fruit arising during a game of Frisbee in a park. In addition, incidental experiences are often those that are fleeting, such as the noted ripeness of the blackberries or other natural features changing with season, weather, or time of day (Tveit et al. 2006). Sensing the ephemeral characteristics of nature can, of course, be a planned motive behind a nature visit, for example visiting nature settings with the intention of observing spring wildflowers, sunrise/sunset, or a migration phenomenon such as cranes moving north in the spring. These events, however, can also make witnessing the unexpected more likely in part based on sensory or aesthetic qualities (Chenoweth and Gobster 1990), for example the experience of contrast such as when sunlight suddenly penetrates a cloudy sky or the discovery of a loud chorus of spring frogs.

Research reveals that routine well-practiced behavior is continually modulated by incidental experience (Wilder et al. 2013). Roth and Jornet (2014) present an etymological exploration of the concept of experience highlighting the importance of the idea that experience, in part, transcends intention. They note the potential for unforeseeable events and outcomes to transform the way people approach the world as a key element of the idea of experience. To better understand this potential for interactions between incidental and intentional nature experience, ideas related to the redirection of attention are considered in the next section.

### The redirection of attention

By drawing upon research of fascination and surprise, we attempt to highlight the potential for redirecting individual attention, thereby fostering transitions between intentional and incidental nature experiences. “Fascination” is described by Hartig et al. (2001) as “effortless attention engaged by objects in the environment or the process of making sense of the environment” (p. 592) and can be a product of either intentional or incidental nature experience. Such experience highlights a transfer of awareness, the effortless shift in attention away from a primary activity, and redirection toward an unplanned focus (Collado and Corraliza 2015; Marselle et al. 2014). Many fascination or discovery experiences have an element of surprise, which means occurring unexpectedly and providing a sudden feeling of wonder or astonishment. A redirection of attention toward an unplanned or unanticipated nature experience (of wonder or interest) may also be understood by considering studies of surprise that emphasize specific physiological and affective responses (Reisenzein et al. 1996; Lindgreen and Vanhamme 2003). Ephemeral experiences are often a source for such redirection, defined in this perspective as nature phenomena that are ever-changing, short-lived, and often seasonal events such as the appearance of a rainbow, the formation of a snowdrift, the blooming of spring wildflowers, or the migration of birds.

The ideas of fascination, discovery, and surprise (collectively, the redirection of attention) are pulled together in a useful way by attention restoration theory or ART (Kaplan 1995). Hartig et al. (2001) paraphrased Kaplan and Kaplan (1989) and described attention restoration as “…situations that involve psychological distance from aspects of one’s usual routines and demands on directed attention (being away), effortless attention engaged by objects in the environment or the process of making sense of the environment (fascination), immersion in a coherent physical or conceptual environment that is of sufficient scope to sustain exploration (extent), and congruence between personal inclinations and purposes, environmental supports for intended activities, and environmental demands for action (compatibility)” (p. 592). Drawing on ART, Berman et al. (2008) describe attention in two ways, involuntary, “…where attention is captured by inherently intriguing or important stimuli …and voluntary…” or directed attention, “where attention is directed by cognitive-control processes” (p. 1207).

These descriptions of attention from ART fit well with the consideration of incidental and intentional experience and how they are supported by green infrastructure. Green infrastructure may be able to create an environment of “being away,” experiences in our daily pattern that take us away (cognitively and affectively) and provide stimulating views, smells, sounds, and sights. Relatedly, incidental experiences within the green infrastructure may also support “being away” via a short-term escape from clock time whose presence dominates daily life (Skår et al. 2010). In addition, green infrastructure provides the potential for both fascination and surprise to redirect our awareness and includes the possibility of making the nature redirection experience a part of our daily life.

### Nudging nature experience

Coupling green infrastructure with the idea of a redirection of attention can also be related to the concept of *nudging*. Nudging in this context refers to guiding the public into nature encounters that they might otherwise not experience. The concept of nudging has received increasing attention as an environmental policy tool for guiding people into more sustainable behavior. Thaler and Sunstein (2008) define nudging as “… any aspect of the choice architecture that alters people’s behavior in a predictable way without forbidding any options or significantly changing their economic incentives. To count as a mere nudge, the intervention must be easy and cheap to avoid. Nudges are not mandates” (p. 6). The aspect of “choice architecture” in the context of green infrastructure planning and incidental nature experience refers to conditions where people do not *necessarily* have to make specific intentional decisions in order to have nature experiences.

### A proposed model for how green infrastructure can counteract extinction of experience

**Fig. 1:**
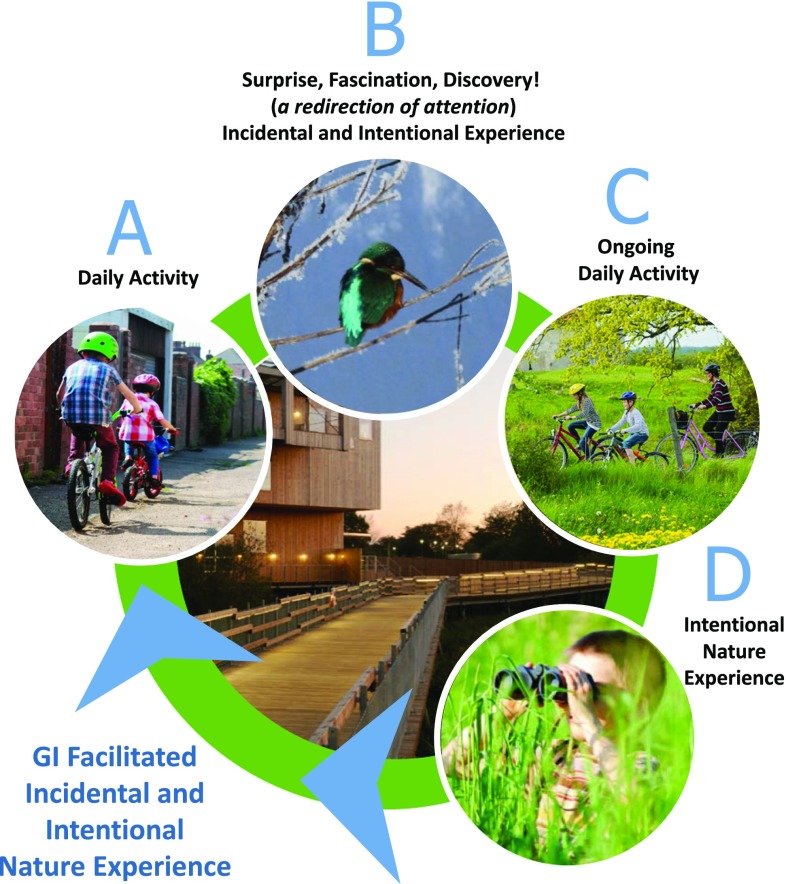
Incidental nature experience cycle

Based on the review in the previous section, we suggest that those nature experiences in which attention is diverted from a primary task and redirected toward nature may have the potential to contribute to individual well-being. Further, we propose that such incidental experience may be able to support the intention for nature experience and may be able to disrupt the trend of diminished contact with nature. Figure 1 represents an experience cycle guided by incidental experience within green infrastructure. There are six main components to the Incidental Nature Experience Cycle and associated transitions, described here (specific examples are provided in the case studies):

1. Daily living activity: a certain amount of daily living activity, defined as the various tasks of daily life, can happen within the green infrastructure. Positive nature experience as a result of daily living activity in the green infrastructure highlights the importance of deliberate design that provides multisensory experience, multiple perspectives, and ever-changing elements (such as seasonality, weather, animal behavior, and vegetative cycles).
2. Redirection of attention: the initial surprise or discovery which may lead to fascination is the nudge or opportunity to experience nature in a new way. Good green infrastructure design may “nudge” or guide the public into nature encounters that they would otherwise not experience.
3. Ongoing daily living activity: this stage in the progression presents the possibility that ongoing daily living may become more oriented toward nature given perceived and experienced benefits.
4. Continued opportunity for incidental experience and related growth of intentional experience of nature. For example, the experiences in green infrastructure may encourage increased use of green infrastructure for meeting daily living needs (transport, fitness, social, etc.) with ongoing accompanying nature experience.

A final note about this proposed cycle: it is important to acknowledge that people have many motivations (and barriers) to nature experience beyond what is modeled in this diagram. For example, in the Kristianstad case study we note the role of social media records of incidental experience that support increased intentional experience.

## Case studies

**Fig. 2:**
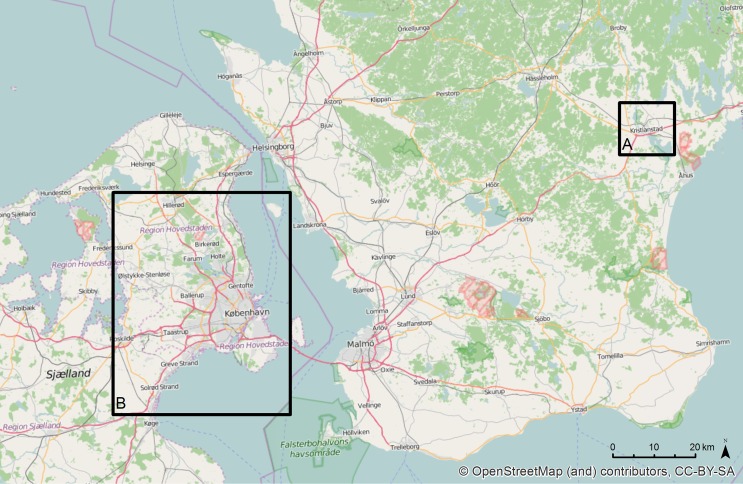
Case study communities within the greater Öresund region: **a** Kristianstad, Sweden, and **b** Copenhagen, Denmark

The following case studies provide examples to support to the use of green infrastructure to facilitate incidental nature experience, such as fascination, discovery, or surprise, or the potential for such, in the context of two communities: Kristianstad, Sweden, and Copenhagen, Denmark (Fig. 2). These urban areas differ in scale and setting yet both are from a Nordic context and should be seen in relation to Nordic characteristics of population density, urban structures, weather conditions, and socio-cultural characteristics.

Kristianstad is located in a heavily agricultural region of NE Scania and has a population of 82 563 residents (Statistics Sweden 2016). Copenhagen is a part of the highly urbanized Öresund area which extends beyond Copenhagen and includes, for example, the Swedish urban municipality of Malmö. Copenhagen has a population of 1.28 m. inhabitants, with 591 481 residents living in the urban municipality (Statistics Denmark 2016). Each of the case studies will present a brief description of local green infrastructure planning, and further one key green infrastructure element in these communities is highlighted to provide an example of the potential to design with incidental nature experience in mind, across various scales and contexts.

### Kristianstad

Most of the Kristianstad municipality corresponds with the lower Helge River watershed, an area of more than 100 000 hectares designated as a UNESCO Biosphere Reserve (Magnusson 2004). The name, “Vattenrike,” translates to “water kingdom” recognizing the ecological and cultural historical significance of the expansive wetlands of the lower Helge River system. Beyond the visitor center and associated educational outreach of the Vattenrike, a strong organizational focus has been placed on providing the opportunity for direct experience of nature as a key aspect of public outreach (Beery and Jönsson 2015). And to be more specific, it is not only the direct experience of nature emphasized by the Kristianstad Vattenrike, but a further emphasis on biodiversity. Phrases such as the following characterize Vattenrike efforts to promote direct experience: “Few places have such a rich and diverse nature as Kristianstad Vattenrike. Here is something for everyone to experience…the best way to learn and understand the landscape’s value is via experiences and knowledge in the places of the Vattenrike” (Vattenriket 2015). The embodiment of this guiding philosophy are the 21 visitor sites established throughout the Biosphere area. The sites form a green network of accessible nature experience opportunity throughout the ecologically significant wetlands. Each site is designed to showcase, protect, or develop one or more of the many socio-ecological phenomena highlighting the importance of the area. In addition, each site provides opportunity for intentional nature experience, for example: signage for nature interpretation, trails for hiking or biking, picnic tables and areas for grilling, docks for fishing, and observation towers for bird watching (Beery and Jönsson 2017). In alignment with the green infrastructure ideal of connectivity (Youngquist 2009), many of the sites are physically connected to other sites or other green spaces via recreation corridors. Bikeways connect eleven of the visitor sites across the biosphere area. Along with the effort to feature the area’s ecological and cultural significance, many of the sites are adjacent to human population concentrations making access for people a key feature. The support of the Vattenrike efforts by the Kristianstad municipality (the Vattenrike Biosphere office is a part of the municipal government structure) allows for close collaboration between the UN designated goals and municipal needs.

**Fig. 3:**
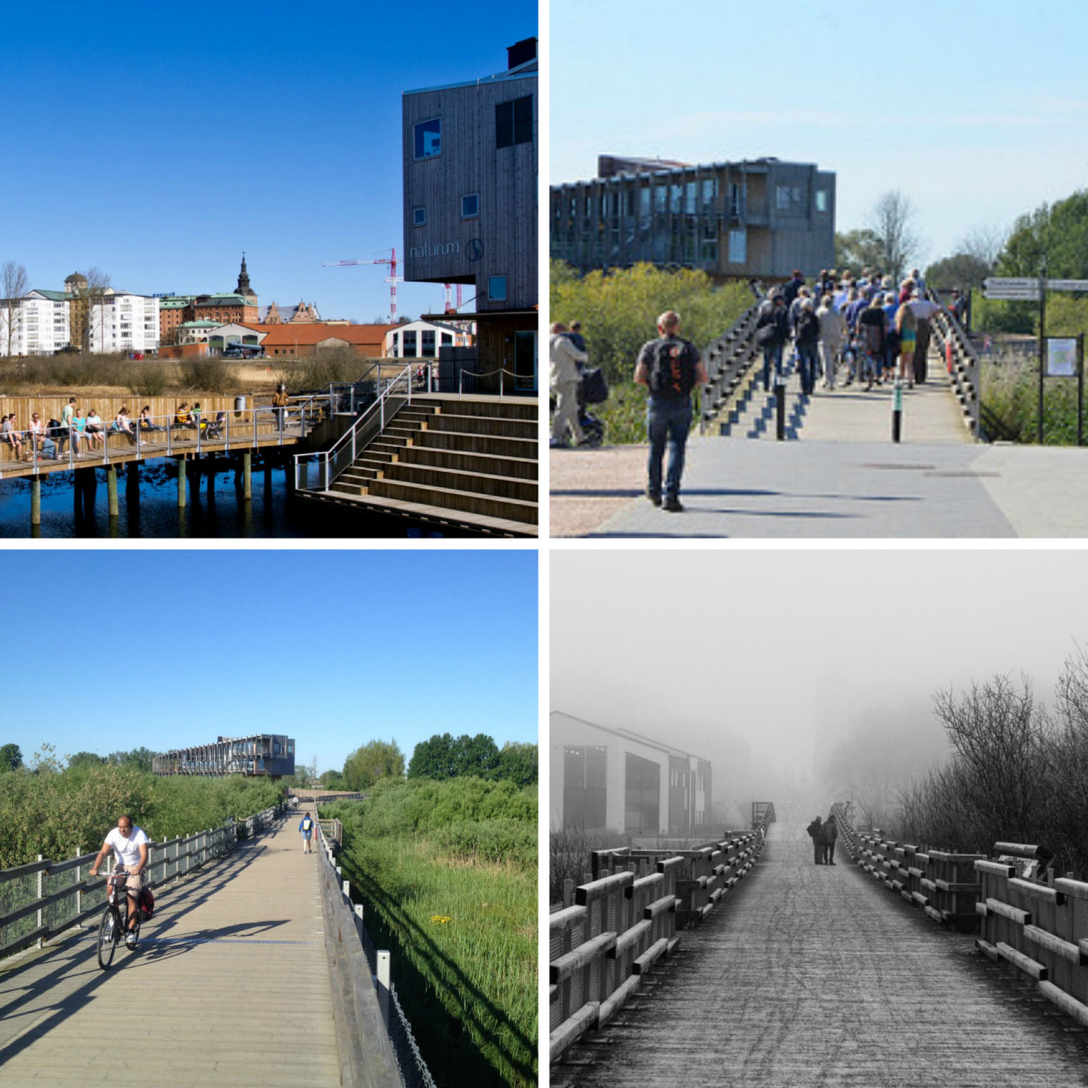
Images of the walk/bike bridge in Kristianstad linking city sections, ecologically significant wetlands, etc.

The bridge spanning the Helge River between the Vattenrike visitor center (Naturum) and Kristianstad city center (Fig. 3) elucidates the role of green infrastructure design in facilitating incidental and intentional nature experience. The bridge is 200 meters long and positioned approximately 3 m over the river surface. Trips over the bridge put users in direct contact with the river and wetlands of the Vattenrike; the corridor over the river and adjacent wet meadows, an expanse of tall wetland reeds managed to allow for seasonal water level fluctuations, provides a direct route between an extensive public parking area and the central business district of Kristianstad. The junction of the bridge and Tivoli Park shore (city center side of bridge) is approximately 200 m from the municipal/regional government offices, 200 m from the train/bus station, 10 m from the community swim center, and 20 m from a group of river frontage apartments. The bridge is used extensively by the public for work, business, and social visits. Bridge use statistics indicate a high volume of traffic; for example, a snapshot sample measurement by the city of Kristianstad between June 26 and July 17, 2015 showed a total of 43 008 foot and bike trips over the bridge, or an average of 2 048 trips per day during this period.^2^

The recent arrival of otter (*Lutra lutra*) in Kristianstad provides an example of how green infrastructure, and the bridge in particular, has the potential to facilitate incidental nature experience. Consider this typical observation from the autumn/winter 2015/2016:

A group of university students was waiting on the outdoor dock/deck structure of the Vattenrike visitor center, perched within the wetlands and attached to the noted bridge. The group was scheduled at the visitor center for an indoor class. Students and instructor were chatting, adjusting clothing to a cold wind, checking phones for messages, etc. (random waiting) when commotion from fish jumping in the water below alerted the group to the arrival of two otters. The otters proceeded to swim around, capturing and consuming fish within 5-10 meters of the student group. This surprising and fast transpiring event redirected attention of the waiting students. There was notable excitement and focused attention on the phenomenon. The event was discussed with enthusiasm, posted on social media and referenced long after the occurrence.

This event provides an example of the incidental nature experience cycle from points A to B in Fig. 1; students engaged in a daily living task (*going to school*) have an incidental nature experience. This incidental and meaningful nature experience was repeated often for many bridge users during the winter 2015/2016. Visitor center staff noted many bridge users experiencing an otter viewing surprise on route to work and these surprise experiences motivated many to return for hopes of further observation (Points C to D on Fig. 1). As word spread (person to person, TV, radio, newspaper, social media), many people made intentional visits to observe the otter; for many, seeing the otter became a social phenomenon as evidenced by social media and direct observation of daily gatherings of residents and visitors. A similar situation had previously been noted regarding overwintering of kingfishers (*Alcedo atthis*) observable from the bridge, and these highly colorful birds surprised and delighted many during the winter of 2014–2015.

The specific location of the bridge in Kristianstad, linking different parts of town, transportation nodes (parking, train, and bus station), and proximity to both city center along with the dynamic quality of the wetlands and river due to regular water level fluctuations, vegetative change, animal behavior, etc. facilitate opportunity for incidental experience. Bridge users have the very real opportunity for surprise, fascination, and attention redirection from their daily living tasks. Further, the extensive and deliberately planned (and connected) green infrastructure of the Vattenrike provides ample opportunity for continued intentional and incidental experience along the many corridors which support daily movement while also addressing conservation efforts designed at supporting a biodiverse ecological community.

### Copenhagen

**Fig. 4:**
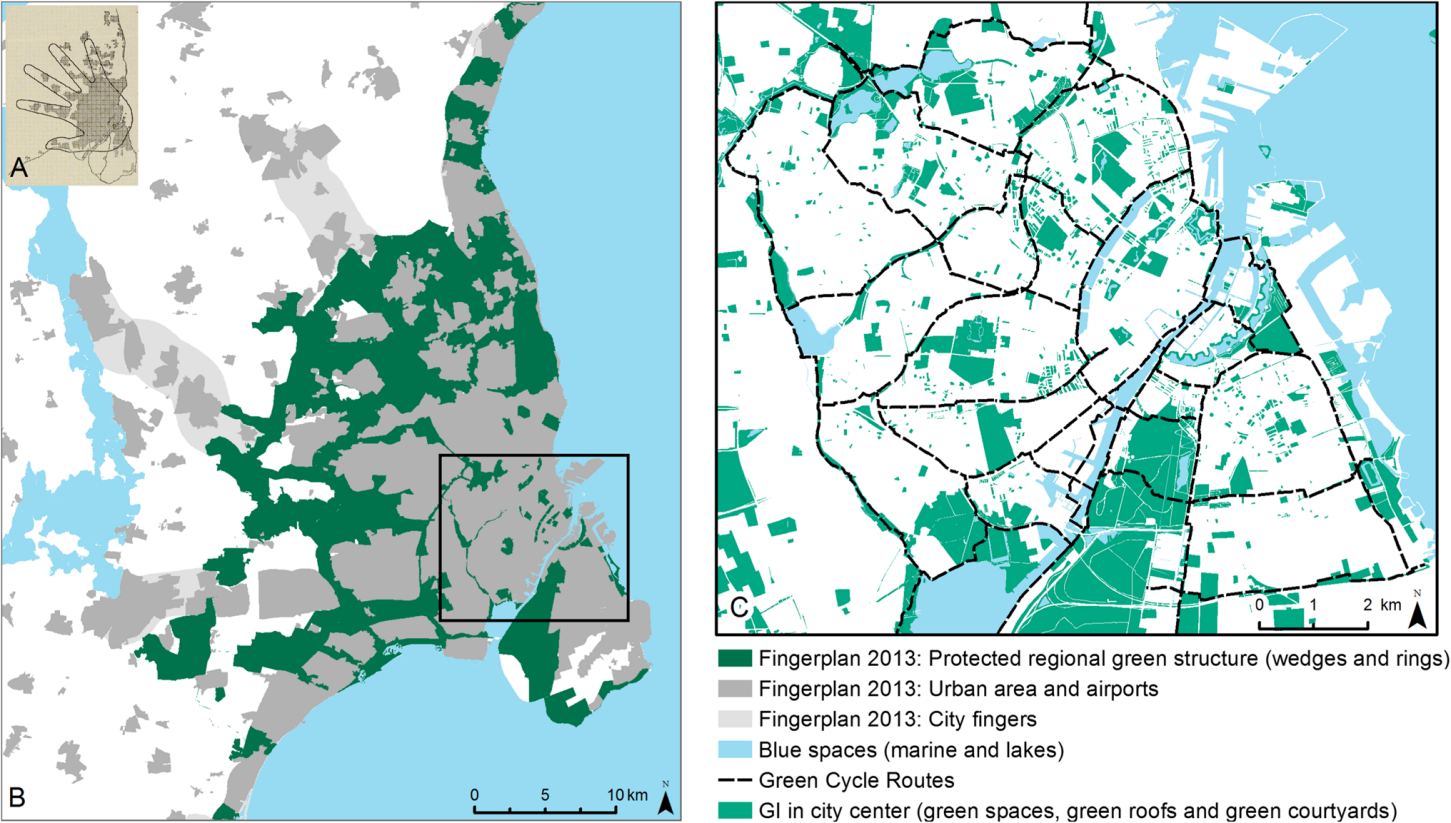
**a** Finger plan from 1947, **b** Copenhagen regional map , and** c** Copenhagen city center

The importance of a green space network providing recreational experience opportunities for the urban population in Copenhagen has been on the planning agenda for many decades. While green infrastructure is not yet implemented as a formal planning approach in Copenhagen, there exists a long planning tradition with focus on green structures. The first coherent green space network plan dates back to 1936 (Forchammer 1936). The plan highlighted the importance of reserving a regional coherent network of green space areas to provide easy close-by access to recreational experiences for the urban population. Most of the plan was realized the following decades (Vejre et al. 2007) and it turned out to be decisive for the ‘Finger Plan’ published in 1947 (Bredsdorff et al. 1947) (see Fig. 4). The Finger Plan was the first regional urban plan in Copenhagen which delimitated the borders of future urban growth while also designating a green infrastructure consisting of green wedges between radial urban fingers along railway and highway infrastructure. The Finger Plan acted as a weak guideline in the following decades, and the green wedges faced rapid urban growth during the economic and population boom in the 1950s and 1960s until strong regional planning was put into power in the 1970s (Caspersen et al. 2006). Since then, controlled urban growth along the radial fingers has occurred in conjunction with an enlargement of the regional green infrastructure via expansion of the green wedges and the introduction of five green rings. Today, the green infrastructure of greater Copenhagen is strongly protected by a national planning act, and the debate of enlargement is ongoing (Ministry of Environment 2015). The outer parts of the green infrastructure are characterized by designated landscapes with a more rural character providing the context for forest recreation and countryside visits, while the inner and more central parts of the green infrastructure have a park character with allotment gardens, and various leisure and outdoor recreation facilities providing for a spectrum of different recreational experience opportunities (Caspersen and Olafsson 2010). The importance of the green infrastructure and related nature experiences are highlighted by a study documenting how arguments of the cultural or intangible ecosystem services linked to recreational experience opportunities rival the other ecosystem services in protection and restoration of two green spaces in Copenhagen (Vejre et al. 2010).

One of the key examples from Copenhagen highlighting the role of green infrastructure design to facilitate incidental and intentional nature experience is the network of green bicycle lanes. Copenhagen is widely known and promoted as a bicycle-friendly city (Pucher and Buehler 2008). A fine meshed network of bicycle lanes provides accessible cycling opportunities in the city (Carstensen et al. 2015), and in 2014, 45% of all journeys to work or education were made by bicycles (City of Copenhagen 2015b). The benefits of cycling include reduced carbon emissions and noise nuisance, while concomitantly improving public health and public urban life. The official planning aim of the city is to increase cycling even more, to make Copenhagen “the best bicycle city in the world” as highlighted in the title of the current bicycle strategy (City of Copenhagen 2011). One of these initiatives toward this aim is a policy focused on making cycling more attractive by a green infrastructure network of green cycle lanes. A green cycle lane is a lane dedicated to cycling along green (and blue) spaces that allow for shortcuts and provide a calm and attractive cycling environment (City of Copenhagen 2015c). The lanes are implemented by making routes through green spaces and constructing missing links such as new cycling bridges crossing busy roads and waters which are linking new parts of the city. In total, 115-km green cycle lanes are planned; by 2015, 58-km lanes were finished. The lanes are mainly focusing on utility cycling (e.g., commuting to work or school) but also provide possibilities for recreational cycling. A recent study in Copenhagen revealed that utility cycling along green and blue spaces is linked to the opportunity for nature experiences. Cyclists were asked to map positive and negative experiences on the daily cycling route and a modeling of the responses highlights the importance of green and blue areas in forming positive experiences for the cyclists (Snizek et al. 2013). Another study made an onsite survey of visitors to an urban nature park and concluded that most visitors were cyclists and that ‘experience nature’ was the most frequent activity while ‘exercise’ and ‘making a shortcut’ were the most frequent main motives for the visit (Jensen 2014). The opportunity to have both a shorter route and access to green space highlights the potential for incidental experience. This example illustrates the proposed incidental nature experience cycle from (A) to (C) in Fig. 1.

The use of social media provides a further example from Copenhagen of the potential of green infrastructure to support incidental experience of nature. The experience of ephemeral phenomena (surprising, inspiring, and/or interesting) related to the natural elements became apparent in a study revealing cultural ecosystem services through Instagram images in Copenhagen (Guerrero et al. 2016). Instagram, a platform for sharing digital images, has millions of users globally, and more than 60 million images are shared everyday (Instagram 2016). The city of Copenhagen encouraged citizens to share images of their city through the hashtag #sharingcph, resulting in thousands of shared images. An analysis of 2 572 geo-referenced images provided by 944 users showed that urban nature was present on 34% all images (Guerrero et al. 2016).

Out of these urban nature images, 27% were focused on ephemeral characteristics of nature, e.g., on green reflections in temporary rainwater puddles, special lights in green spaces, water surfaces, or sunsets/sunrises. Another 10% of the urban nature images were focused on ‘spontaneous’ nature, that is, e.g., urban wildlife, wild plants, weeds, insects, and fungus appearing spontaneously in a city (Guerrero et al. 2016). Hence, one-third of the shared images were captured in an instant with a mobile phone, which documents peoples’ appreciation of ephemeral nature related to the elements, wildlife, and wild plants appearing spontaneously in the city. These images contribute to scenes of mystery and surprise and again highlight the importance of incidental dimensions of nature experiences. This sharing of experience provides a tangible example of social components of incidental and intentional nature experience, i.e., social interaction of ‘sharing,’ ‘liking,’ and ‘following’ each other’s images in the Instagram e-community.

## Discussion

The bridge in Kristianstad and the bicycle routes in Copenhagen provide examples of quality green infrastructure. The examples demonstrate the important potential for green infrastructure to support incidental experience of nature and emphasize the integration of green infrastructure into the urban setting as a way to create opportunity, to facilitate, guide, or *nudge* nature experience. Ultimately, incidental and non-intentional nature experience may be able to play an increasingly important role addressing concerns regarding a diminished nature experience, the noted extinction of experience. The remainder of this discussion focuses on incidental nature experience implications for green infrastructure planning and research.

### Green infrastructure planning

Cities across the world are investing in the provision, management, and enhancement of public green spaces as a result of the growing evidence of the link between nature experience and human well-being outcomes (Mitchell and Popham 2008; Kardan et al. 2015). New green infrastructure planning strategies are frequently recommended to address findings on the links between nature experience, public health, and well-being. For example, recent studies have urged landscape planners to develop innovative strategies for encouraging access to quality green spaces for different durations and frequencies of nature experience given that these different doses are varyingly associated with different health outcomes (Shanahan et al. 2016). We highlight the planning implications of considering different types of nature experience, including incidental experience. Our examples from Kristianstad and Copenhagen show that it is possible to introduce green and blue elements to improve the nature experience or heighten interest or awareness while simultaneously serving accessibility to daily living tasks. We acknowledge the challenge of planning for incidental experience and, however, emphasize that deliberate planning efforts should take seasonality, weather, animal behavior, vegetative cycles, biodiversity, refuge, etc. into account. The green infrastructure examples from Sweden and Denmark illustrate such possibility and provide consideration across a range of urban scales that may be transferable across much of Europe, and perhaps beyond. One aspect of consideration related to intentional and/or incidental nature experience is the findings that have suggested that urban living conditions (in general) may undermine human well-being (specifically, mental health), while conditions in rural areas may support it (Peen et al. 2010). Hartig and Kahn (2016) considered this question of urban versus rural setting impact on human well-being and noted a broad range of environmental factors that can impact well-being. They suggested that it may be more productive to consider the specific factors of concern (such as population density, air quality, transportation options, etc.) versus simply dichotomizing urban versus rural.

Specific recommendations about how green infrastructure could be designed to support a range of intentional and incidental nature experiences are presented in Table 2. The functional design categories of access, corridors/routes, vegetation, and earthen structure have been chosen in order to present examples of potential general categories/subcategories. Note, the suggestions presented in Table 2 are not comprehensive and in many cases may overlap and/or be highly intertwined with each other. For example, vegetation for biodiversity, structural interest, wildlife food, wildlife shelter, human refuge, and viewing at close range could all be a part of one particular design element.

**Table 2:** Recommendations for the integration of incidental nature experience design elements into landscape planning for daily nature experience opportunity

Green infrastructure		
Design category	Design attribute	Design purpose
Access	Water	Sensory experience of water via route proximity, bridges, docks, etc.
	Views	Opportunity to look beyond the immediate, or to gain a protected view—overlooks, outlooks (observation towers), blinds, etc.
	Wildlife	Structures to enhance wildlife habitat, e.g., nest boxes and platforms in proximity to human experience^a^
	Furnishings	Placement of public chairs and benches for human enjoyment and relaxation proximate to water, vegetation, and views
Connectivity	Mobility	Nature-rich routes for human mobility (note that wildlife corridors are another aspect of green infrastructure planning with different priorities—this focus is upon human access to nature)
Vegetation	Structural variety	Variation in plant size, shape, texture, growth pattern, etc.
	Biodiversity	Variation in species and species distribution
	Seasonal interest	Plant cycle variation (e.g., blooming vegetation, fruiting vegetation, seed variation and availability, autumn color, winter weeds, winter fruit, etc.)
	Wildlife	Vegetation to enhance conditions for a diversity of wildlife* (providing food and shelter)
Earthen structure	Refuge	Refuge created via use of topographic structure to eliminate distracting noise or views or to separate areas of conflicting land use (buffering function)

^a^Accommodations to protect wildlife well-being must be considered in conjunction to human proximity; for example, the sensitivity of nesting for many species demands careful consideration

### Human experience

The exploration of incidental nature experience highlights the need for green infrastructure strategy and planning to emphasize human experience. One way in which human experience may be able to be emphasized is via a consideration of scale (Beery and Jönsson 2017). Gobster et al. (2007) contend that “it is difficult for people to understand, care about, and act purposefully upon phenomena that occur at scales beyond our direct experience” (p. 960) and refers to a landscape scope of human experience as a meaningful scale. Colding and Barthel (2013) note the impact of a meaningful scale using the idea of cognitive resilience building: “the perceptions, memory, and reasoning that people acquire from frequent interactions with local ecosystems, shaping peoples’ experiences, world views, and values toward local ecosystems and ultimately toward the biosphere” (p. 162).

Another way in which human experience may be able to be emphasized in green infrastructure strategy and planning is via existing efforts to ensure that human environments are resilient to environmental, social, and economic challenges (EU 2013). For example, when EU strategy (EU 2013) presents green infrastructure as capable of the absorption of excess water from heavy rains as an alternative to building flood protection and further note that such effort that could also enable walking and cycling opportunity, the focus is on flood control and reduction of carbon emissions. We argue, however, that in addition to these important objectives, direct human experience of nature must also be highlighted. Good green infrastructure can offer both urban resilience and public opportunity for regular and meaningful experiences of nature.

### Future research

Earlier in this paper, we proposed that those nature experiences in which attention is diverted from a primary task and redirected toward nature has the potential to contribute to individual well-being. Further, we proposed that such incidental experience may support the intention for nature experience and may be able to disrupt the trend of diminished contact with nature. Using diagrams and case studies, we attempted to build support for these ideas; however, empirical testing is needed. Also needed is a research agenda for green infrastructure which enables a systematic assessment of the relationship between multiple types of structure, nature experience, and links to human well-being. We have taken an inductive approach in this perspective in order to initiate and stimulate further discussions about the role of incidental nature within green infrastructure planning. We wish to promote a research plan, however, to investigate these ideas empirically. For example, active living research (Sallis et al. 2016) and the review of green infrastructure and human health presented in Tzoulas et al. (2007) provides strong examples of how the study of incidental nature experience and daily living fit as part of a broad-based and coordinated effort to support better understanding of human well-being. The specific ideas in this perspective article can be tested via a review by municipal planners in order to gain insights into the feasibilities of the suggested measures. In addition, municipal planners may be able to provide a deeper sense of values attached to green infrastructure in order to guide future planning on behalf of nature experience. Ultimately, we propose that empirical efforts may be able to provide information to address the extinction of experience in a structured and useful way.

## Concluding reflection

The poem by Nobel Laureate Tomas Tranströmer at the beginning of this paper describes how the emergence of early spring flowers is enchanting (or “spellbinding”). Tranströmer described an unexpected ephemeral nature experience easily missed if not for chance movement and a contrast of color that served to redirect attention and transported the observer to some new mental place, as noted later in the poem: “…the wind-flowers open a secret passage to the real celebration…” (Tranströmer 1983, p. 20). Perhaps this celebration noted by Tranströmer is our human relationship with more than human nature? We argue in favor of making the possibility for such experience a part of how we think about green infrastructure.

## Footnotes

1 We recognize that nature is a constructed concept and acknowledge the growing literature that seek alternatives to human–nature dualistic thinking (Haraway 2008; Castree 2014). Here we define nature as “an organic environment where the majority of ecosystem processes are present (e.g., birth, death, reproduction, relationships between species). This includes the spectrum of habitats from wilderness areas to farms and gardens” (Maller et al. 2006, p. 46; Keniger et al. 2013). This particular definition is of interest as it spans the “spectrum” from the largely nonhuman to the heavily human. Similarly, Bratman et al. (2012) describe nature as “areas containing elements of living systems that include plants and nonhuman animals across a range of scales and degrees of human management, from a small urban park through to relatively “pristine wilderness”” (p. 120). Both of these definitions move us toward a more integrated and relational understanding of nature–culture and avoid complete opposition, or separation of human and nonhuman elements.

2 Carl Almström. Traffic engineer Kristianstad Municipality, email response 10 February, 2016.
